# Supplementary material for: Mechanisms governing the pioneering and redistribution capabilities of the non-classical pioneer PU.1
Source: Nat Commun. 2020 Jan 21;11:402. doi: 10.1038/s41467-019-13960-2 (PMC6972792; doi:10.1038/s41467-019-13960-2)
Supplement: Supplementary file 7 — Source data [file 41467_2019_13960_MOESM7_ESM.zip › Source_Data/Figure2/Figure2F_MetascapeResults/Enrichment_PPI/hits_MCODE_ALL_PPIColorByCluster.pdf]

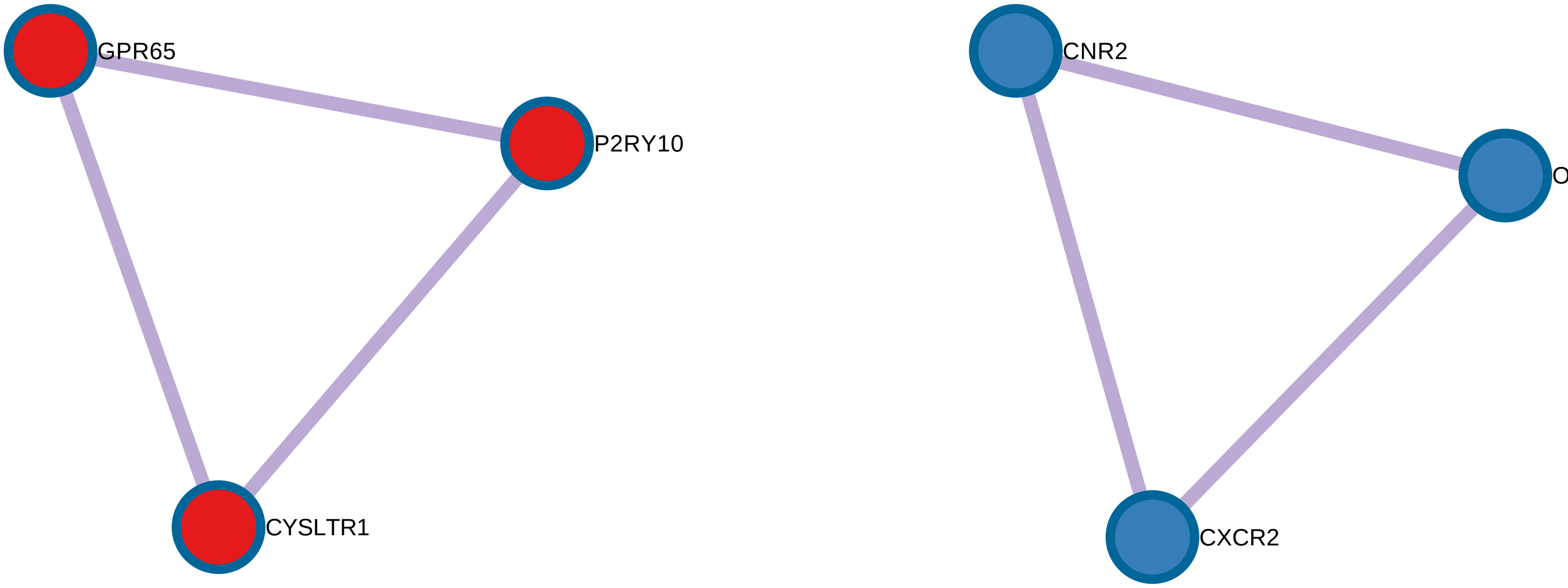
GPR65
P2RY10
CYSLTR1
CNR2
CXCR2

MCODE1
MCODE2

created by
http://metascape.org
